# The Application of Polyethersulfone Ultrafiltration Membranes for Separation of Car Wash Wastewaters: Experiments and Modelling

**DOI:** 10.3390/membranes13030321

**Published:** 2023-03-10

**Authors:** Wirginia Tomczak, Marek Gryta

**Affiliations:** 1 Faculty of Chemical Technology and Engineering, Bydgoszcz University of Science and Technology, 3 Seminaryjna Street, 85-326 Bydgoszcz, Poland; 2 Faculty of Chemical Technology and Engineering, West Pomeranian University of Technology in Szczecin, ul. Pułaskiego 10, 70-322 Szczecin, Poland

**Keywords:** car wash wastewater, cleaning agent, fouling, membrane cleaning, ultrafiltration

## Abstract

The wastewater generated as a result of car washes is considered a new source of water. However, recovered water must meet the required quality criteria for reuse. For this purpose, the ultrafiltration (UF) process can be successfully used. The main aim of the present work was to investigate the influence of the membrane’s molecular weight cut-off (MWCO) on the UF performance in terms of the fouling phenomenon and retention degree of car wash wastewater. Moreover, for a better understanding of the fouling mechanisms, Hermia’s model was used. The experimental studies were conducted with the use of two polyethersulfone (PES) membranes (MWCO of 10 kDa and 100 kDa). It has been noted that the used membranes provided a high-quality permeate and excellent turbidity removal, up to 99%. Moreover, it has been noted that the MWCO membrane has a significant impact on the fouling mechanism. Generally, a much greater intensity of fouling for the membrane with MWCO of 100 kDa was observed. Results obtained in the present study showed that both real wastewaters and the clean solutions used for washing cars cause the fouling phenomenon. It has been proven that rinsing the membranes with water is not sufficient to recover the initial membrane’s performance. Hence, periodic chemical cleaning of the membranes was required. Fitting the experimental data to Hermia’s model allowed us to indicate that membranes with MWCO of 100 kDa are more prone to intermediate blocking. To sum up, the findings suggest that for the UF of the car wash wastewater, the use of membranes with MWCO equal to 10 kDa is recommended.

## 1. Introduction

Nowadays, the car wash industry is growing rapidly all around the world. It has been estimated that approximately 200 L of water is consumed in automatic wash services for each car [1,2,3]. Consequently, car washing generates a large volume of wastewater which presents a complex composition and is extremely damaging to the environment. Indeed, car washes discharge effluents contain significant concentrations of various contaminants, such as detergents, oil, grease, dust and sand, chemicals, heavy metals, as well as organic matter [4,5,6]. As a result, wastewaters are characterized by high values of both the chemical oxygen demand (COD; >400 mg O_2_/L) [2,7] and turbidity (up to 1000 NTU) [2]. Furthermore, it should be noted that solutions used for washing cars contain a lot of chemicals and surfactants, hence, pose a serious threat to the environment [8]. Importantly, as outlined in a recently published review paper [9], the treatment of car wash wastewater is considered a new source of water. Indeed, effective recycling of wastewater allows us to reduce the consumption of freshwater [10].

Obviously, water recovered from wastewater must meet the required quality criteria for reuse [2,11,12,13,14]. For instance, suspensions may be effectively removed from wastewater by several methods, such as coagulation, sedimentation, adsorption and sand filtration [3,7,15,16,17,18,19]. It is necessary to point out that car wash wastewaters also contain significant amounts of pathogenic microorganisms [20,21,22]; hence, ensuring biosecurity is another key challenge. In the literature, there is general agreement that, for the pre-treatment of wastewater, ultrafiltration (UF) technology can be successfully used.

Several studies documented the application of UF for the separation of carwash wastewater with the use of both ceramic [23,24] and polymeric [10,25,26,27,28] membranes. However, since the cost of manufacturing ceramic membranes is relatively high [9,29,30,31,32], polymeric ones are advantageous for industrial applications. According to Susanto et al. [33], polyethersulfone (PES) is the most often used as the UF membrane material. Indeed, ultrafiltration PES membranes have been successfully used in a wide range of applications. It is related to the fact that the principal characteristics of PES are: high thermal, chemical and oxidation stability, hydrolytic strength and good mechanical properties [34,35,36]. Car wash wastewaters contain a wide range of different compounds, which may increase their potential negative impact on the polymeric membrane [37]. For this reason, in the present study, UF experiments using various types of wastewater were conducted.

It is well known that fouling is the most challenging issue for the application of membrane technology in wastewater treatment. This complex phenomenon is caused by the adsorption of the feed compounds on the membrane surface and/or its pores through several mechanisms, mainly complete blocking, standard blocking, intermediate blocking and formation of the cake layer [38]. As a result, a significant decrease in the membrane’s performance is observed. Hence, it is obvious that the fouling leads to increased operational costs, reduced treatment efficiency and declined process productivity [19,39,40,41,42]. The effectiveness of surfactant removal using UF membranes may be affected by several factors, for instance, membrane and surfactant characteristics, and the presence of other pollutants and process conditions [43]. In order to maintain high filtration performance, regular membrane cleaning is required. It is widely considered that reversible fouling can be removed by rinsing membranes with water, while irreversible fouling requires the use of chemical agents [12]. With regard to polymeric membranes, a key challenge is to determine whether the cleaning agents cause rapid destruction of the polymer structure [37].

It must be recognized that UF membranes are characterized by a wide range of pore sizes which has a key impact on UF efficiency [43,44,45]. In this context, the goal of this work was to investigate the influence of the membrane’s molecular weight cut-off (MWCO) on the UF performance in terms of the fouling phenomenon and retention degree of car wash wastewater. For this purpose, commercial PES membranes were used. In addition, for a better understanding of the fouling mechanisms, a mathematical model was applied. To the best of the authors’ knowledge, the present paper is the first to demonstrate the use of the model established by Hermia [38] for analyzing the permeate decline during the UF process of car wash wastewater. In addition, the intensity of the PES membranes fouling by pure cleaning agents was investigated.

## 2. Materials and Methods

### 2.1. Ultrafiltration Unit

In the present study, 2 types (UE10 and UE50) of commercial ultrafiltration PES membranes were used. The membranes were manufactured by TriSep Corporation (Goleta, CA, USA). Table 1 summarizes the used membrane’s characteristics.

**Figure 1:**
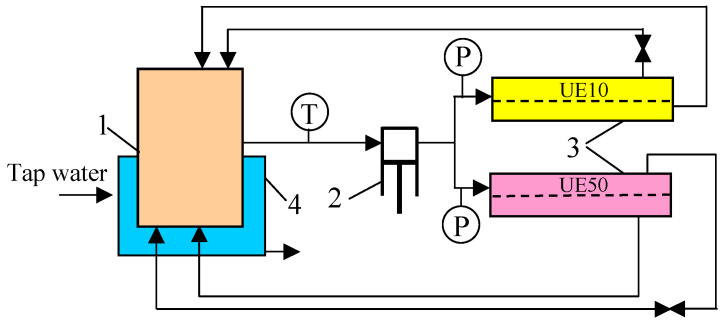
UF installation with two plate modules. 1—feed tank, 2—pump, 3—module, 4—water cooling, T—thermometer, P—manometer.

The UF experiments were conducted using the installation presented schematically in Figure 1. The membranes were mounted in plate modules with an active area of 24 cm^2^. The module channel (height of 0.7 mm) was filled with a polypropylene mesh. The modules were powered by a 3CP Stainless Steel Plunger Pump model 3CP1221 (CAT PUMPS, Hampshire, England). The studies were carried out at the transmembrane pressure (TMP) of 0.2 MPa. The applied feed flow velocity was equal to 0.6 or 1 m/s. The feed (volume of 2 L), after flowing through the module, was returned to the tank. Changes in membrane permeability were measured for distilled water at the TMP in the range from 0.05 to 0.3 MPa. Permeate samples were collected for 10 min with an accuracy of 0.2 mL, which resulted in a measurement error of less than 1 L/m^2^h. During the investigations, the feed temperature was between 293 and 295 K, which was obtained by cooling the feed tank with tap water. Prior to the measurement series, the membranes were compacted at a TMP of 0.2 MPa for 2 days.

The volume concentration ratio (VCR) was determined according to the following equation:

(1) $$VCR=\frac{V_{F}}{V_{F}-V_{P}}$$

where V_F_ [L] is an initial feed volume, and V_P_ [L] is the volume of the collected permeate.

### 2.2. Feed Solutions

In order to investigate the separation properties of the ultrafiltration PES membranes, nonionic (Triton X-100, Merc, Darmstadt, Germany) and anionic (SDS, Sigma Aldrich, Shanghai, China), surfactant solutions were used as a feed. In addition, the studies were performed for synthetic wastewater prepared from a mixture of commercial car wash agents, the composition of which provided by the manufacturer (EuroEcol, Łódź, Poland) is presented in Table 2. Throughout this paper, the following terms for tested cleaning agents were used: white (Euro Turbo Foam), blue (Euro Turbo Foam Color Blue), green (Euro Active Foam Green) and wax (Hydrowax).

With regards to the practical use of the above-mentioned cleaning agents, it should be indicated that the process of car washing involves several stages. After pre-rinsing, the car is washed with surfactant solutions (white, blue or green). Subsequently, it is rinsed with the reverse osmosis (RO) permeate, and finally, the car body is waxed with the use of a solution named wax. The fluids from each of the stages flow into the settling tank, where they are mixed. In order to successfully implement the concept of water reuse, the resulting mixture can be treated by the UF process. In the present study, the commercial surfactant concentrates (Table 2) were diluted with the nanofiltration (NF) permeate to the concentration of 0.5% (wax to 0.2%), which corresponds to the concentration values of solutions used at car washes. Worthy of note, the NF permeate (60 µS/cm) obtained from tap water (590 µS/cm) was also used for washing the installation and rinsing the membrane modules.

As indicated above, the purpose of the present work was to investigate the impact of pure cleaning agents on the membrane fouling phenomenon. In turn, to determine the effect of real wastewater, the solution obtained during car washing with 0.5% Euro Turbo Foam solution was used. The solution (5 L) was applied with a sponge to the car body, and then, the dirty solution was collected in a tank. Subsequently, the wastewater was sedimented overnight, and the upper layer (2 × 2 L) was used for the UF studies.

### 2.3. Analytical Methods

The studies of membrane morphology and composition of deposits formed on the membrane’s surface were performed using a Hitachi SU8000 Field Emission Scanning Electron Microscope (FESEM; Hitachi, Tokyo, Japan). All the samples were sputter-coated with chromium.

The particle size distribution in the studied solutions was determined with the use of a Mastersizer 3000E instrument (Malvern Panalytical Ltd., Malvern, UK).

The surfactants concentration was determined using a HACH vial digestion solution for anionic (LCK432), cationic (LCK 331) and nonionic (LCK 334) surfactants. The values were recorded automatically with the use of a Hach Lange DR2800 spectrophotometer (Hach Lange, Düsseldorf, Germany).

The turbidity of tested solutions was measured with a portable turbidity meter model 2100 AN IS with a detection limit of 0.01 NTU (Hach Company, Loveland, CO, USA).

The membrane hydrophobicity was determined by water contact angle (WCA). The sessile drop method using the Contact Angle System OCA (Data Physics, Filderstadt, Germany) apparatus was used for the WCA measurements. The applied volume of the water drop was equal to 10 μL.

### 2.4. Modeling

Mathematical modeling plays an important role in determining the mechanisms of flux decline during membrane processes [46]. In the present study, in order to reveal the fouling mechanisms during the treatment of car wash wastewaters with the use of ultrafiltration PES membranes, a mathematical model established by Hermia [38] was applied. The motivation lies in the fact that this model has been successfully used to investigate the membrane fouling phenomenon in UF processes of various wastewaters types, such as oily wastewaters [47,48,49], industrial wastewaters [50] and cold-rolling emulsion wastewaters [51].

Hermia’s model is expressed by the following equation [38]:

(2) $$\frac{d^{2}t}{dV^{2}}=k{\left(\frac{dt}{dV}\right)}^{n}$$

where t is the filtration process time, V is the permeate volume, k is the constant, and *n* is the characteristic exponent depending on the fouling mechanism, which includes: cake formation (*n* = 0), intermediate blocking (*n* = 1.0), standard blocking (*n* = 1.5) and complete blocking (*n* = 2.0; Table 3).

A more detailed description of the modeling methodology was presented in our previous studies [49,52].

## 3. Results and Discussion

### 3.1. Properties of Washing Agents and Membranes Characteristics

The washing agents used as a feed solution contained various substances with the properties of surfactants; however, their composition is not provided by the manufacturers. Hence, in order to determine the concentration of non-ionic, anionic and cationic surfactants, the Hach cuvette tests were performed. The obtained results are shown in Table 4. The concentration of non-ionic and anionic surfactants in the solutions was in the range of 0.47–0.77 g/L. Importantly, cationic surfactants were not found in the samples analyzed. This finding is satisfactory since this type of wastewater compound is very toxic in the environment [53,54,55].

**Figure 2:**
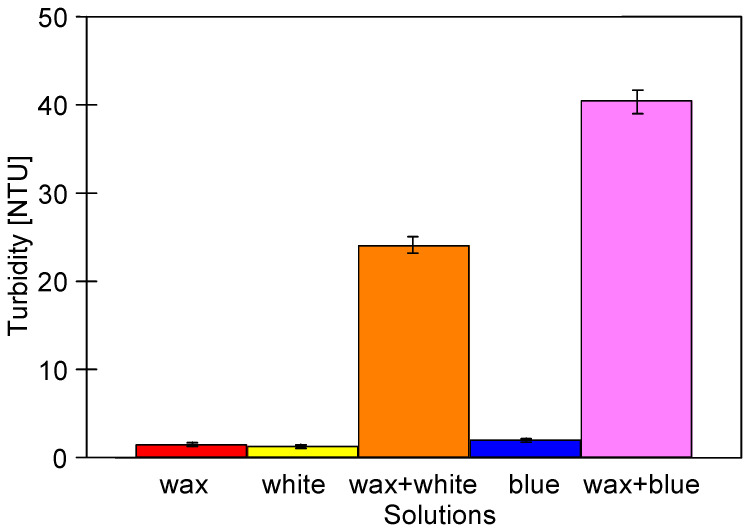
Turbidity of cleaning agents solutions. Concentrates: 0.2% (wax), 0.5% (white), and 0.5% (blue).

The cleaning agents that have been used in the present study are well soluble in water. However, the performed analysis has shown that their solutions (0.5%) differed significantly (Figure 2). Indeed, the noted values of turbidity were equal to 1.3 NTU (wax), 1.1 NTU (white) and 1.9 NTU (blue). After washing the car with foam and rinsing it with water, the car’s body is waxed. Then, the wax solution flows into the collected wastewater. To replicate this procedure, a wax solution was added to the surfactant solution (white or blue), which led to a slightly increased in the mixture turbidity. Specifically, the turbidity of the mixture of wax and white solutions was equal to 24 NTU, while that of the mixture of wax and blue solutions was over 40 NTU. This finding indicated that a mixture of pure cleaning agents could cause membrane fouling during the UF process. As it has been mentioned above, the detailed composition of individual cleaning agents is the secret of manufacturing; however, it can be assumed that the interactions of various polymer and wax components (Table 2) caused the noted increase in turbidity. In addition, the surfactant adsorption on the UF membrane’s surface may cause fouling [33,43]. For this reason, in the present study, various mixtures of these agents were used as feeds. The turbidity of the real wastewater after the sedimentation was equal to 82.1 NTU.

**Figure 3:**
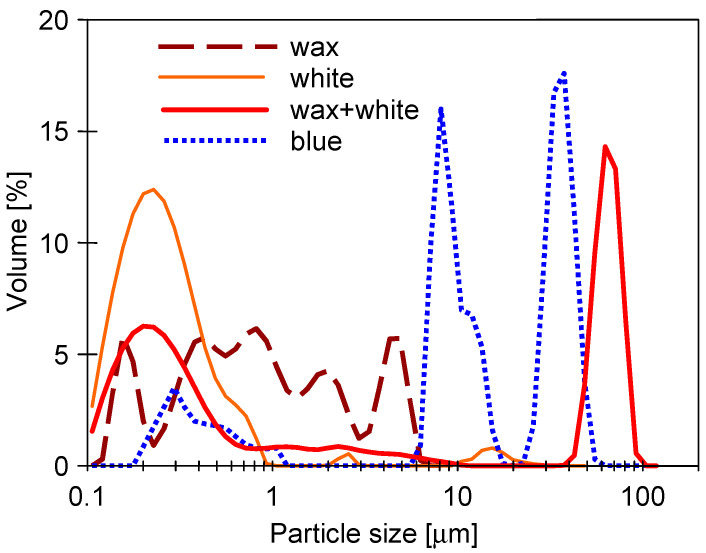
Distribution of particle size in cleaning agents solutions.

Analysis of the particles’ size distribution has shown that the increase in the mixture’s turbidity was caused by the increase in the number of larger particles. For instance, the white agent solution contained mainly particles below 1 μm, while the wax solution was characterized mostly by particles below 6 μm. However, in a mixture of these agents, particles over 50 μm were present (Figure 3). Worthy of note, the solution of the blue agent contained much larger particles than its white counterpart, which contained no dye.

It should be pointed out that although the turbidity of feed solutions was in the range of 20 to 42 NTU (synthetic wastewaters), the performed UF process ensured the high-quality permeate. Indeed, it has been found that the turbidity of obtained permeate was equal to 0.1–0.2 NTU and 0.3–0.5 NTU for the UE10 and UE50 membranes, respectively. With regards to the real wastewater (82.1 NTU), a permeate characterized by a turbidity of 0.48–0.52 NTU was obtained. According to the aforementioned data, it can be clearly indicated that the used membranes provided excellent turbidity removal, up to 99%. Obviously, this finding can be attributed to the fact that the pores of UF membranes are much smaller than the particle present in the feed (Figure 3). This result is in complete agreement with observations presented in [25], wherein it has been shown that the cellulosic UF membrane with MWCO of 100 kDa used for the treatment of car wash wastewater allowed to obtain the permeate of 0.64 ± 0.02 NTU.

It is well known that the UF process is less effective for the retention of low molecular weight substances. With regard to surfactants, the retention degree depends on their type and concentration [56,57]. However, to be complete, it should be noted that after exceeding the critical micelle concentration (CMC), the formed micelles become larger, which facilitates their retention by UF membranes [43,45]. Accordingly, in [45], the reported retention degree of sodium dodecylbenzenesulfonate (SDBS) by PES membrane with MWCO of 10 kDa was more than 50%. In the present study, it has been found that the UE10 and UE50 membranes provided a significantly lower degree of surfactant retention (Table 5). For instance, the SDS retention was equal to 28% and 15% for UE10 and UE50 membranes, respectively. In the case of the white agent, the retention values were equal to 40% (the UE10 membrane) and 42% (the UE50 membrane). Moreover, it has been found that the addition of the wax solution resulted in increased turbidity of the feed, as a result of which more intensive fouling of the membranes improved separation properties. Consequently, higher retention degree, equal to 60% (UE10 membrane) and 53% (UE50 membrane) was noted. In turn, for both of membranes tested, the total permeability for Triton was noted.

Based on the findings presented above, it should be pointed out that PES membranes have weak hydrophobic properties, which resulted in both increased retention of anionic surfactants (e.g., SDS) and permeability to nonionic surfactants (e.g., Triton) [43,45].

### 3.2. UF Process of Synthetic Wastewaters

**Figure 4:**
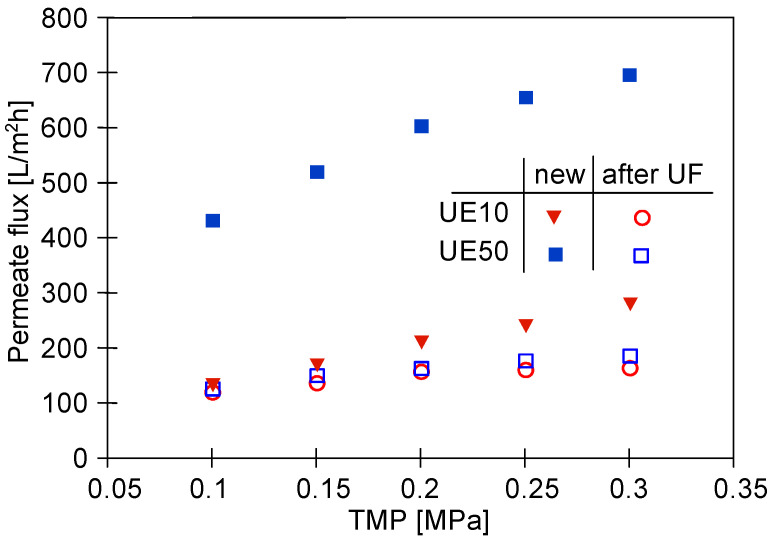
The impact of TMP value on the obtained permeate flux. Feed: distilled water.

The water permeability for the new UE10 and UE50 membranes is shown in Figure 4. Importantly, nearly three times higher permeate flux was noted for the UE50 membranes. Indeed, for instance, at a TMP of 2 MPa, the water flux was equal to 600 L/m^2^h and 208 L/m^2^h for the UE50 and UE10 membranes, respectively. Undeniably, this observation is related to the fact that UE50 membranes are characterized by a 10-times higher value of MWCO compared to UE10 membranes (Table 1).

**Figure 5:**
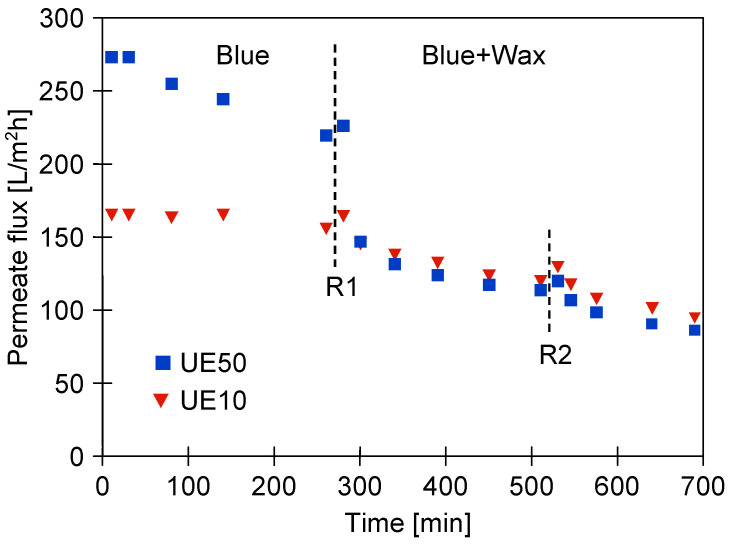
Changes in the permeate flux during UF of blue and blue + wax solutions. R1, R2—modules rinsed with water. TMP = 0.2 MPa, feed flow velocity: 0.6 m/s.

Firstly, the experimental studies on the UF performance were conducted with the use of the blue cleaning agent (Figure 5). The process was performed under TMP and feed flow velocity of 0.2 MPa and 0.6 m/s, respectively. During the first 10 min of the process run, a significant decline in the permeate flux was observed. Indeed, for UE50 and UE10 membranes, the flux decreased from 600 L/m^2^h to 272 L/m^2^h and from 208 L/m^2^h to 165 L/m^2^h, respectively. This finding substantiates previous findings in the literature [57], wherein it has been shown that detergent solution significantly reduces the flux during the UF process. According to [43], this observation can be attributed to the adsorption of surfactants on the membrane surface. Importantly, the results of this experiment demonstrated the difference in the flux profile for the two membranes tested. Indeed, contrary to the UE10 membrane, the permanence of the UE50 membrane was not stable. It decreased throughout the process run, and finally, the flux was equal to 219 L/m^2^h. It can be explained by the fact that the tested solution was characterized by a large amount of 10–50 µm particles that could penetrate into the pores of the UE50 membrane. Hence, for this membrane, a different fouling mechanism occurred. This conclusion was confirmed by the results obtained by applying Hermia’s model (Section 2.4). Indeed, the findings showed that the best fit to experimental data for the UE50 and UE10 membranes corresponded to the intermediate blocking and cake layer formation, respectively (Table A1, Appendix A). For intermediate pore blocking, particles block the membrane pores and, additionally, attach to the surface of the membrane. In turn, in the case of the cake layer mechanism, particles do not block the membrane pores; however, they accumulate on the membrane surface, forming permeable multilayers [58,59]. According to the aforementioned data, it can be concluded that the membrane MWCO has a significant impact on the fouling mechanism during the separation of cleaning agents during the UF process. In addition, it should be mentioned that since internal fouling is difficult to remove, rinsing the modules with water did not restore the initial process performance. It led to only a slight increase in the permeate flux (Figure 5, point R1).

In the next stage of the process, the wax solution (0.2%) was added to the blue agent solution, which led to an increase in the solution turbidity from 2 to 41 NTU. This, in turn, resulted in the enhancement of the fouling intensity. The contamination of the membranes increased with the process run, and, as a result, the permeate flux systematically decreased (Figure 5). Finally, similar performance values were obtained for the two membranes tested. Worthy of note, fitting the experimental data to Hermia’s model revealed that during the UF process of the blue mixture, the formation of a cake layer was the key reason for the flux decline for both membranes used (Table A1, Appendix A). Hence, it can be assumed that the intermediate mechanism above reported for the UE50 membrane occurred mainly in the first minutes of the process run. Rinsing the membranes with water (point R2) did not allow us to restore the initial membrane’s performance. As a result, after 700 min of the process, the permeate flux decreased to the level of 90 L/m^2^h. In Figure 4 (‘after UF’), it can be seen that the maximum performance of both membranes decreased to a similar level. This result confirmed the much more intensity of the fouling in the case of the UE50 membrane.

**Figure 6:**
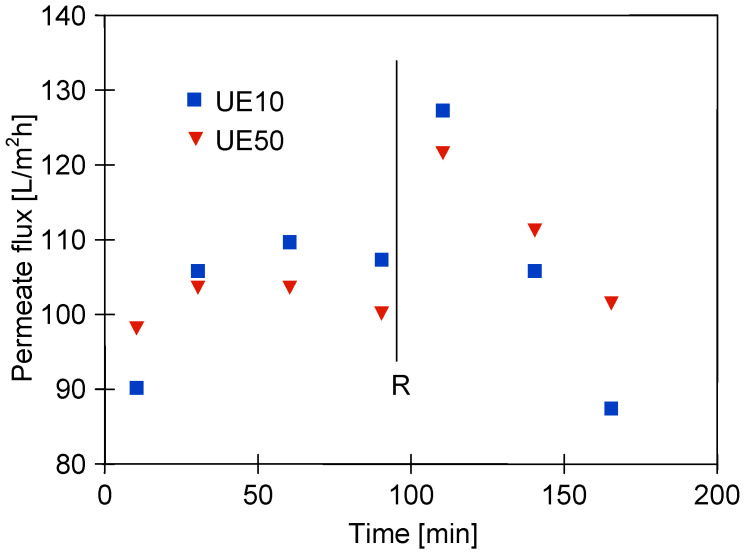
Changes in the permeate flux during UF of blue + wax solution. R—modules cleaning is presented in Figure 7. TMP = 0.2 MPa, feed flow velocity: 1 m/s.

In the literature, there is general agreement that the formation of deposits on the membrane surface and the thickness of the gel layer depends on the feed flow rate [60,61]. In the present study, after completing the tests presented in Figure 5, the feed flow velocity was increased from 0.6 to 1 m/s, which significantly increased the permeate flux (Figure 6, first 100 h). This result confirmed that increasing the turbulence of the flow decreases the polarized layer thickness, and consequently, the amount of deposit formed on the membrane’s surface was reduced [25]. However, the permeate flux obtained for the UE50 membrane was still almost two times lower than the initial value presented in Figure 5. It is worth mentioning that this finding is in accordance with the results presented in [25], wherein the treatment of carwash wastewater by MF and UF membranes has been investigated. In the above-mentioned study, it was shown that the increase in flow rate from 0.5 to 1.5 L/min led to an increase in the performance of hollow fiber polyetherimide membrane with a mean pore diameter of 0.4 μm.

The use of the wax agent during car washing aims to apply a wax protective layer on the car body. It can be assumed that a layer of waxes is also formed on the surface of UF membranes, which causes the permanent fouling phenomenon and a decline in the permeate flux (Figure 5). It should be mentioned that washing cars has the purpose of removing not only dirt but also old layers of wax. Undoubtedly, cleaning solutions contain compounds that facilitate this task. For instance, in the case of the green agent (Table 2), Sodium Cumenesulphonate has such a property. It is an anionic surfactant used in liquid and powdered detergent formulations, heavy-duty cleaners, wax strippers, and dishwashing detergents. Since it is used as a solubilizer and cloud point depressor in wax, it was assumed that it could also be successfully used for cleaning membranes. Taking the abovementioned into account, in the next stage of the present study, membrane cleaning was investigated.

**Figure 7:**
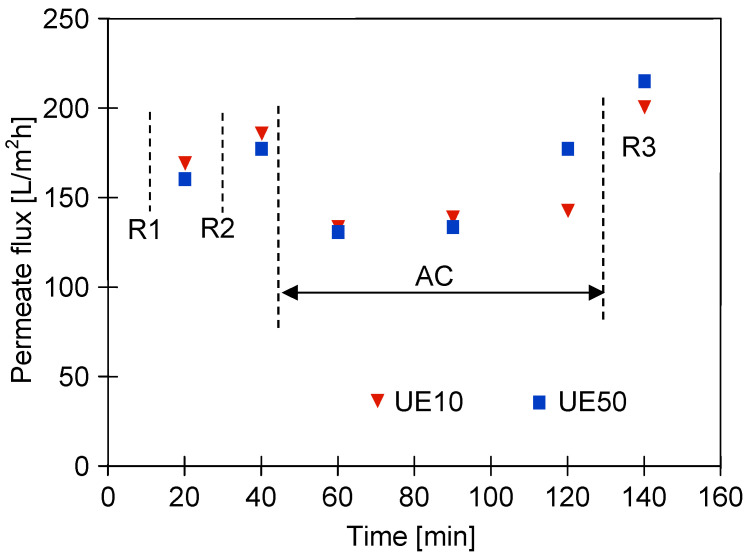
Changes in the permeate flux during cleaning of membranes contaminated with components of the blue + wax solution. R1, R2—membranes rinsed with NF permeate and soaked in water for 12 h, AC-filtration of the green solution, R3—flushing the installation with the NF permeate.

Changes in the permeate flux during cleaning membranes contaminated with components of the blue + wax solution are shown in Figure 7. In the first stage, the membranes were rinsed with the NF permeate, and then, the modules were immersed in water (osmotic rinsing) for 12 h (points R1 and R2). As a result, the permeate flux of the UE10 membrane increased to 185 L/m^2^h, which was similar to the initial value reported (Figure 4). However, such an effect was not obtained for the UE50 membrane, which confirmed that its pores were significantly blocked. Subsequently, the filtration of the green solution (0.5%) was performed for 90 min, which allowed us to obtain a slight increase in the permeate flux, especially for the UE50 membrane. This finding indicated that the flow of the green solution through the membrane could dissolve the wax present in its pores. After flushing the installation with the NF permeate, the maximum flux for UE10 and UE50 membranes was equal to 200 L/m^2^h and 214 L/m^2^h, respectively. Hence, it can be concluded that the initial performance of the UE10 membrane was restored, while the flux of the UE50 membrane was still significantly lower than that reported for the virgin membrane (Figure 4). In addition, it was observed during the experimental investigation that restarting the separation process of the blue + wax solution led to the reduction in the permeate flux to the level reported before the membrane cleaning (Figure 6, after 100 min) within 60 min.

**Figure 8:**
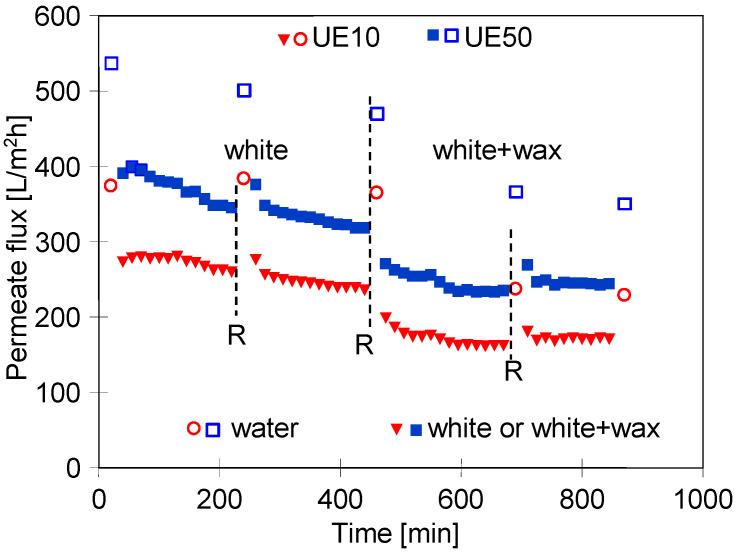
Changes in the permeate flux during UF of white solution and white + wax mixture. R—modules rinsing with water. TMP = 0.2 MPa, feed flow velocity: 1 m/s.

**Figure 9:**
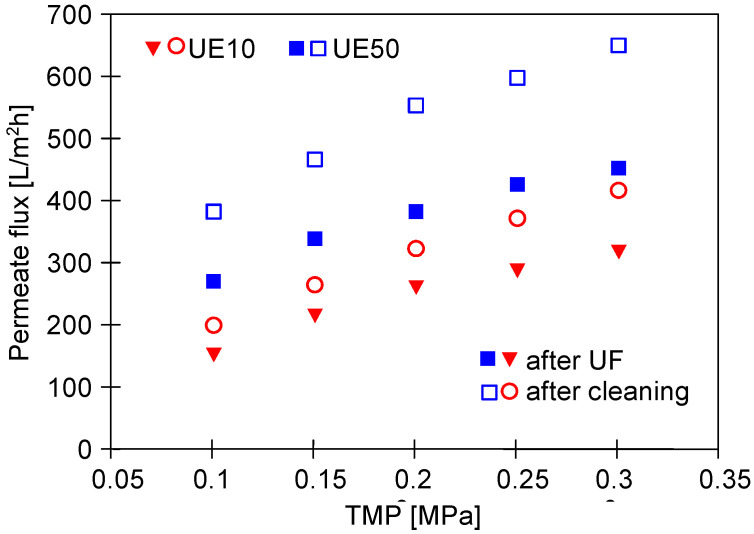
Impact of the TMP on the maximum permeate flux for the membranes after separation of the white + wax mixture (Figure 8) and after membranes cleaning with NaOH solution (0.5 g/L) for 2 h.

Different results in terms of process performance obtained for blue and green agents indicated that the type of cleaning agent used as a feed has a significant impact on the fouling intensity. In other studies [43,62], a decrease in the permeate flux in the range of 20–80% was reported, depending on the surfactant type. To confirm the above, in the next measurement series, the UF experiments were carried out using as a feed the white + wax mixture, which was characterized by two times lower turbidity compared to the blue + wax one (Figure 2). As expected, the addition of wax to the surfactant solution significantly reduced the permeate flux (Figure 8, from 450 h). However, the decline in the process performance was much lower than that observed during the UF process of the blue + wax mixture (Figure 5). Moreover, it has been found that the permeate flux for the UE50 membrane decreased only twice compared to the initial value and was much higher than that noted for the UE10 membrane. Between the measurement series, the membranes were rinsed with water, and changes in the maximum flux (points R) were measured. It has been found that after feeding the modules with the white + wax mixture, the flux decreased; however, it quickly stabilized at the levels of 350 L/m^2^h and 230 L/m^2^h for UE50 UE10 membranes, respectively. To be complete, it should be indicated that fitting the experimental data to Hermia’s model indicated that during each stage of the process run, the formation of a cake layer was the dominant fouling mechanism for both membranes used (Table A2, Appendix A). Rinsing the membranes with sodium hydroxide (NaOH) solution (0.5 g/L) for 2 h allowed us to obtain a flux similar to the initial values noted for the clean membranes (Figure 9). It can be explained by the fact that NaOH solution hydrolyses surfactant molecules, which leads to a decrease in emulsion stability and, consequently, facilitates their removal from the membrane surface [43,62,63,64]. Hence, it can be concluded that the results obtained in the present study are in complete agreement with those presented in the literature. Indeed, NaOH solutions have been successfully used for cleaning UF membranes fouled with different types of wastewater, for instance, oily waste [65] and dairy wastewater [66].

### 3.3. Wastewaters Concentration by UF process

**Figure 10:**
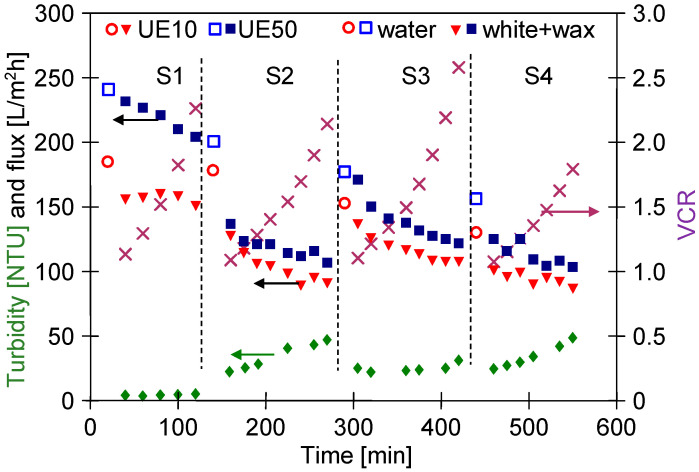
Changes in the feed turbidity, permeate flux and VCR during the UF process with continuous permeate collection. S1–S4—measurement series. Feed: S1—wax (0.2%) + white (0.5%), S2–S4: white (0.5%) + wax (0.2%). Feed flow rate: 1 m/s. Water—permeate flux after rinsing the membranes with water.

The results presented and discussed in the previous section were obtained for experiments performed at a constant feed concentration. For this purpose, the permeate was returned to the feed tank. However, with regard to the water reuse strategy, the obtained UF permeate is collected, which leads to an increase in the solute concentration in the feed. Figure 10 shows changes in the feed turbidity, permeate flux and volume concentration ratio (VCR) during the UF process of white and wax solutions with continuous permeate collection.

The addition of wax to the detergent solution increased the turbidity (Figure 2). The turbidity of the wax+white mixture was similar to that determined for unmixed components and was 2.3 NTU. In the S1 series, the feed was concentrated (VCR = 2.25), which resulted in an increase in the feed turbidity to 5.14 NTU. Over 100 min of the UF process run, the permeate flux for the UE10 membrane was stable; however, for the UE50 membrane, it significantly decreased from 232 L/m^2^h to 204 L/m^2^h. It can be attributed to the fact that the wax and white solutions contain particles below 10 µm (Figure 3) which could block the larger pores of this membrane. This assumption has been confirmed by the results obtained by fitting the experimental data to Hermia’s model (Table A3, Appendix A). Indeed, it has been determined that for the UE50 membrane, the fouling was primarily (the first 120 min) governed by complete blocking and then by a cake layer formation. In turn, the flux decline during the UF with the UE10 membrane, the flux decline was caused mainly by the formation of a cake layer. Worthy of note, for complete pore blocking, the particles deposit on the membrane surface and block the entrance of pores [67].

**Figure 11:**
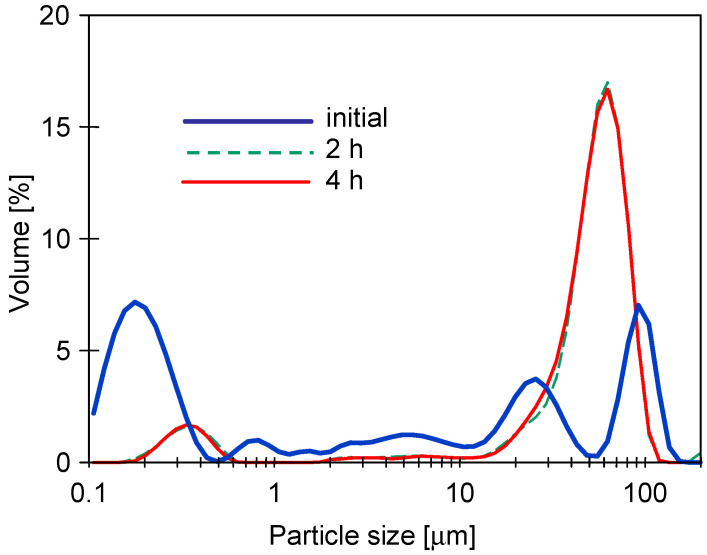
Changes in particle size in the white + wax mixture during the UF process.

In the subsequent experimental series, the process was investigated with the white wax mixture used as a feed, which was characterized by higher turbidity (23–24 NTU). Performing the UF process of the feed characterized by higher turbidity resulted in a greater decrease in permeate flux during the initial 60 min, after which the flux stabilized (Figure 10 (S2–S4)). Analysis of the particle size distribution performed during the concentration of the white + wax mixture demonstrated that the particles dispersed in the feed increased in size during the process (Figure 11). Interestingly, despite the continuous increase in the feed concentration, the particle size distribution after 2 and 4 h of the process run was similar. This noteworthy result indicated that particles agglomeration in the installation occurred rapidly (e.g., due to an increase in the pressure and mixing—mutual collisions), and the amount of suspended matter stabilized during the UF, most likely as a result of the particles settling on the membranes surface and the installation walls.

After each measurement series, the membranes were rinsed with water. However, it did not prevent a decrease in the maximum permeate flux (Figure 10). Indeed, it has been reported that during the separation of four feed portions, the flux decreased by 23% and 43% for UE10 and UE50 membranes, respectively. This finding indicates that the during the feed concentration, the fouling phenomenon will be more intense than during the UF of the diluted feed (Figure 8). Hence, additional chemical cleaning of membranes is required.

**Figure 12:**
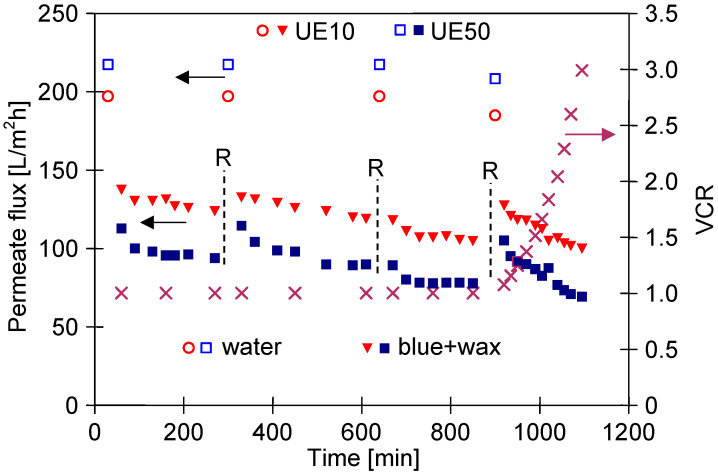
Changes in the permeate flux and VCR during the UF process. Feed: the blue + wax mixture, periodic rinsing (30 min) with green solution (points R). The continuous permeate collection from 900 min.

To gain further insight, in the next stage of the present study, the separation of the blue + wax solution was carried out with periodic cleaning of the membranes with the use of the green agent solution. The results shown in Figure 12 confirm that periodic cleaning significantly reduced the permeate flux decline. A greater decrease in flux has been noted during the feed concentration (from 900 min UF), which indicated more intense fouling, and consequently, effective membrane cleaning is a challenge.

**Figure 13:**
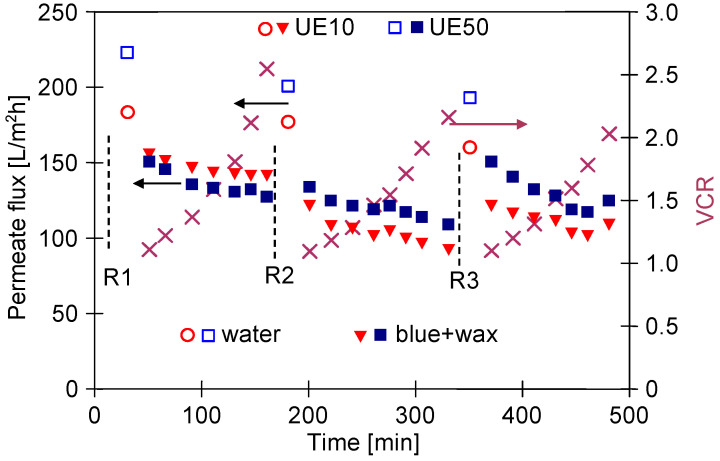
Changes in the permeate flux and VCR during the UF process with continuous permeate collection. Feed: the blue + wax mixture, periodic rinsing with green solution (point R1, R2-60 min) and R3 (120 min).

After completing the measurement series presented in Figure 12, the time of membranes rinsing with the green solution was increased from 30 to 60 min (Figure 13). It has been found that it allowed us to recover the values of maximum flux to 183 L/m^2^h and 225 L/m^2^h for the UE10 and UE50 membranes, respectively (Figure 13, point R1). However, the recorded flux values after repeated concentration runs systematically decreased, which indicates that the intensity of the fouling increased. Undoubtedly, the extension of the cleaning procedure time ensured more effective cleaning of the UE50 membrane’s surface. Indeed, finally, the permeate flux for this membrane was slightly higher than that obtained for the UE10 membranes. Worthy of note, analysis performed with the application of Hermia’s model showed that during each stage of the above-mentioned measurements series (Figure 12 and Figure 13), the cake layer was the main reason for the flux decline for two membranes tested (Table A4 and Table A5, Appendix A).

### 3.4. UF Process of Real Wastewaters

**Figure 14:**
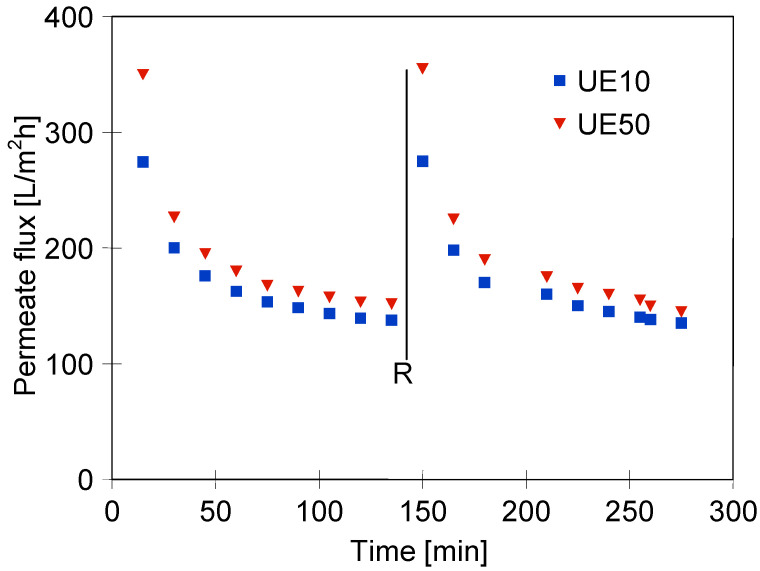
Changes in the permeate flux during the UF process of real wastewaters. Feed: the solution collected during car washing with 0.5% solution of the white cleaning agent. TMP = 0.2 MPa, feed flow velocity: 1 m/s. R-rinsing using 1 g/L NaOH.

**Figure 15:**
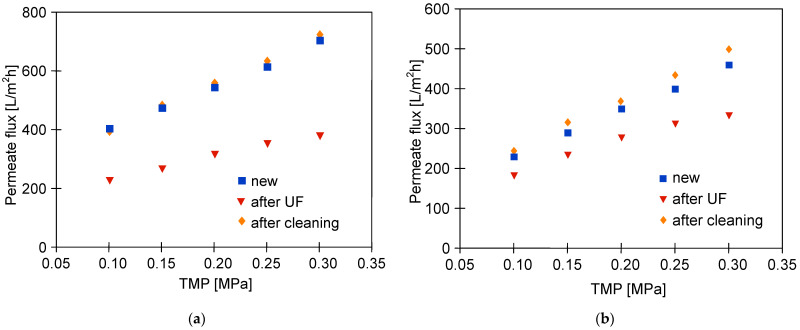
Impact of the TMP on the maximum permeate flux for the membranes after separation of the real wastewaters and after membranes cleaning with NaOH solution (1 g/L) for 2 h. (**a**) UE50, (**b**) UE10.

In the last stage of the investigation, wastewater obtained during car washing with the withe solution was used as a feed in the UF process. As indicated earlier, during the filtration of the pure white solution, the permeate stabilized at the level of 350 L/m^2^h and 280 L/m^2^h for the UE50 and UE10 membranes, respectively. As expected, during the UF process of real wastewater, a greater flux decline was noted (Figure 14). In addition, it has been determined that the difference in the membrane’s performance was less significant. Indeed, after 135 min of the process run, the flux of the UE10 was equal to 137.5 L/m^2^h, while for the UE50 membrane, it was 156.7 L/m^2^h. These findings clearly confirm that the contaminants removed from the car caused the membrane fouling phenomenon. Hence, it should be pointed out that membrane cleaning is of great importance. The results shown in Figure 15 indicate that the use of alkaline cleaning agents is an effective method. Indeed, it has been reported that for both modules used, the initial membrane’s performance was restored. Worthy of note, in the next measurement series, the permeate flux of regenerated membranes decreased in a similar way as during the first series.

Finally, it should be pointed out that fitting the experimental data to Hermia’s model allowed us to indicate that during the UF process of real wastewater, the formation of a cake layer was the dominant fouling mechanism for both membranes used (Table A6, Appendix A). Based on this finding, it can be assumed that although different values of the permeate flux for UE10 and UE50 membranes were obtained, the dominant fouling mechanism was not influenced by the average membrane pore size.

### 3.5. Membranes Fouling

**Figure 16:**
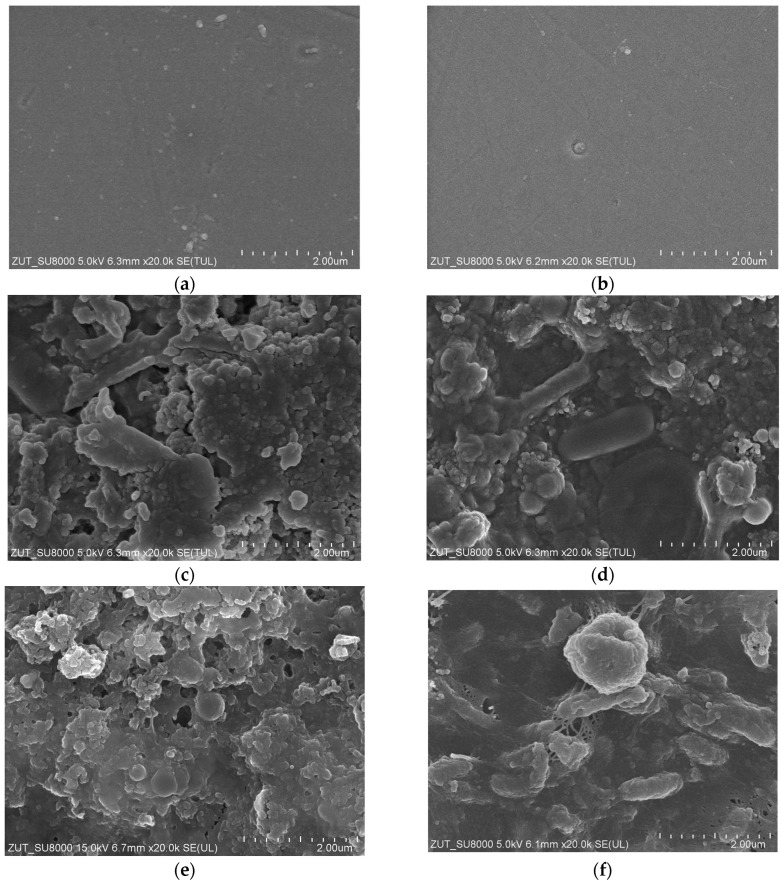
FESEM images of the membranes’ surface: (**a**) Virgin UE10; (**b**) Virgin UE50; (**c**) UE10 with deposits after separation of the blue + wax solution; (**d**) UE50 with deposits after separation of the blue + wax solution; (**e**) UE10 with deposits after separation of the real wastewater; (**f**) UE50 with deposits after separation of real wastewater.

After completing the measurement series shown in Figure 13 and Figure 14, the membranes were rinsed with water, and their samples were analyzed with the use of a scanning electron microscope. Roughly speaking, it has been confirmed that during the UF of the detergents and waxes mixtures, a deposits layer was formed on the membranes’ surface. Worthy of note, the deposit on the UE10 membrane surface (Figure 16c) was less compact, and the pores in its structure were visible. In turn, the deposit formed on the UE50 membranes was less porous (Figure 16d), which significantly hindered the feed flow. Most likely, this finding was the reason that the decrease in maximum performance determined for the UE50 membranes was much greater than that noted for the UE10 membrane (Figure 4).

**Figure 17:**
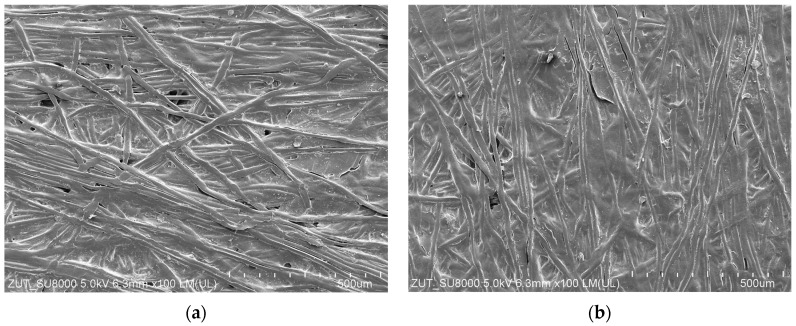
FESEM images of the membrane support surface: (**a**) Virgin UE50; (**b**) UE50 after separation of the blue + wax solution (Figure 12).

The solutions tested in the present study may affect the membrane material causing its degradation [37]. However, the SEM studies have proven no damage to both the active and support layers of the membranes. Figure 17 shows SEM images of the UE50 membrane before and after the separation of the blue + wax solution.

## 4. Conclusions

The application of UF technology for the treatment of car wash wastewater was investigated. The evidence from this study confirmed that for this purpose, PES membranes could be successfully used in terms of both process performance and permeate quality. It has been noted that the used membranes provided a high-quality permeate. Indeed, excellent turbidity removal, up to 99%, was obtained.

The results obtained in the present study clearly confirmed that car wash wastewater causes significant membrane fouling. However, pure cleaning agents used for washing the cars also led to the permeate decline during the UF process. This finding can be explained by the fact that the solutions contain a large number of various ingredients that create a mixture of surfactants and waxes. As a result of their interactions, the turbidity of the solution increased, which caused significant fouling of the membranes.

The use of alkaline cleaning solutions (NaOH solution) allowed us to remove deposits from the membrane’s surface, and, as a result, the initial module’s performance was restored. It has been determined that the solutions used in the present work did not damage the PES membranes tested. However, to determine the membrane’s chemical resistance, longer tests should be conducted.

The membrane MWCO has a significant impact on the permeate flux decline. Generally, a much greater intensity of fouling for the membrane with MWCO of 100 kDa was observed. Fitting the experimental data to Hermia’s model allowed us to indicate that these membranes are more prone to intermediate blocking. In addition, it has been proven that rinsing the membranes with water is not sufficient to completely remove the fouling phenomenon. Hence, periodic chemical cleaning of the membranes is required. High cleaning efficiency can be demonstrated by some agents used for car washing, such as in the case of the Euro Turbo Active Green liquid.

Undoubtedly, the present study provides the framework for the application of ultrafiltration PES membranes for the treatment of car wash wastewater. The obtained results suggest that for this purpose, the use of membranes with MWCO equal to 10 kDa is recommended.

## Figures and Tables

**Table 1:** Characteristics of ultrafiltration PES ultrafiltration membranes used in the present study (manufacturer data). WCA (water contact angle)—measured using the sessile drop method.

Membrane	MWCO [kDa]	pH Range	Hydraulic Permeability [Lm^2^h/bar]	WCA [°]	Thickness [μm]
UE10	10	2–12	74	73 ± 1.3	160–200
UE50	100	2–12	296	68 ± 2.2	160–200

**Table 2:** The composition of the cleaning agents concentrate.

Trade Name	Name Used in the Present Study	Component	Concentration [%]
Euro Turbo Foam	white	diethylene glycol butyl ether	1–5
		benzenesulfonic acid	1–5
		polymers	1–5
Euro Turbo Foam Color Blue	blue	blue dye	
		diethylene glycol butyl ether	1–5
		benzenesulfonic acid	1–5
		polymers	1–5
Euro Turbo Active Green	green	green dye	
		sulfonic acids, C14-17-sec-alkane, sodium salts	2.5–5.0
		ethylenediaminetetraacetic Acid Tetrasodium Salt	2.5–5.0
		sodium Laureth Sulfate	<2
		sodium N-(2-carboxyethyl)-N-(2-ethylhexyl)-beta-alaninate	<2
		amines, C12-14-alkyldimethyl, N-oxides	<2
		sodium Cumenesulphonate	<2
Hydrowax	wax	polymeric waxes	
		diethylene glycol monobutyl ether polymer	10–20

**Table 3:** Fouling mechanisms based on Hermia’s model.

Fouling Mechanism	*n*	Physical Concept	Schematic Description
Cake formation	0	solutes diameter is larger than membrane pores size, solutes deposit on the particles that already block the pores	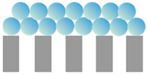
Intermediate blocking	1.0	a single particle can precipitate on other particles to form multi-layers	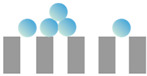
Standard blocking	1.5	particles are adsorbed and deposited on the internal pore wall	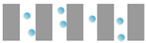
Complete blocking	2.0	solutes diameter is similar to the size of membrane pores, and consequently, the number of open pores is reduced	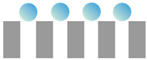

**Table 4:** The concentration of surfactants [mg/L] in the tested solutions of cleaning agents (0.5%).

Surfactants	White	Blue	Green
non-ionic	470 ± 10	560 ± 24	520 ± 18
anionic	770 ± 4	620 ± 8	650 ± 5

**Table 5:** Surfactant retention [%] by the PES membranes. Feed concentration: SDS (130 mg/L), Triton X-100 (500 mg/L), waste: white (0.5%) + wax (0.2%).

Membrane	SDS	Triton	White (Anionic)	Waste (Anionic)
UE10	28 ± 1	0	40 ± 1	60 ± 2
UE50	15 ± 2	0	42 ± 3	53 ± 2

## Data Availability

The data presented in this study are available on request from the corresponding author. The data are not publicly available due to the institutional repository being under construction.

## Appendix A

**Table A1:** Coefficient of determination (R^2^) Hermia’s model. The UF process with the constant feed concentration (Figure 5).

Feed	UF Time [min]	Complete Blocking		Standard Blocking		Intermediate Blocking		Cake Formation	
		UE10	UE50	UE10	UE50	UE10	UE50	UE10	UE50
blue	0–260	0.3472	0.3815	0.3592	0.3436	0.3719	0.6210 *	0.3987	0.4923
blue + wax	280–690	0.9118	0.7862	0.8915	0.8359	0.8723	0.9085	0.9030	0.9121 *

* Indicates the best-fitting model.

**Table A2:** Coefficient of determination (R^2^) Hermia’s model. The UF process with the constant feed concentration (Figure 8).

Feed	UF Time [min]	Complete Blocking		Standard Blocking		Intermediate Blocking		Cake Formation	
		UE10	UE50	UE10	UE50	UE10	UE50	UE10	UE50
white	0–220	0.4949	0.7047	0.5117	0.7354	0.5290	0.7652	0.5644 *	0.8200 *
white	240–440	0.5275	0.4782	0.5045	0.5548	0.5316	0.5827	0.5871 *	0.6390 *
white + wax	460–670	0.4255	0.4330	0.4699	0.4733	0.5166	0.5158	0.6028 *	0.6144 *
white + wax	690–870	0.3553	0.4416	0.3580	0.4524	0.3606	0.4634	0.3654 *	0.4856 *

* Indicates the best-fitting model.

**Table A3:** Coefficient of determination (R^2^) Hermia’s model. The wastewater concentration by the UF process (Figure 10).

Feed	UF Time [min]	Complete Blocking		Standard Blocking		Intermediate Blocking		Cake Formation	
		UE10	UE50	UE10	UE50	UE10	UE50	UE10	UE50
white + wax	0–120	0.5099	0.9873 *	0.5128	0.9856	0.5156	0.9838	0.5212 *	0.9793
	140–270	0.7803	0.6464	0.8144	0.6803	0.8443	0.7136	0.8883 *	0.7753 *
	290–420	0.8898	0.7218	0.9041	0.7666	0.9172	0.8090	0.9395 *	0.8826 *
	440–550	0.6723	0.7682	0.6896	0.7836	0.7062	0.7975	0.7360 *	0.8205 *

* Indicates the best-fitting model.

**Table A4:** Coefficient of determination (R^2^) Hermia’s model. The wastewater concentration by UF process (Figure 12).

Feed	UF Time [min]	Complete Blocking		Standard Blocking		Intermediate Blocking		Cake Formation	
		UE10	UE50	UE10	UE50	UE10	UE50	UE10	UE50
blue + wax	0–270	0.4972	0.4696	0.5160	0.4974	0.5357	0.5270	0.5772 *	0.5881 *
	300–610	0.4903	0.5143	0.5176	0.5634	0.5462	0.6146	0.7150 *	0.6066 *
	640–850	0.4774	0.4211	0.4977	0.4418	0.5193	0.4644	0.5650 *	0.5120 *
	900–1095	0.6771	0.6248	0.7211	0.7199	0.7632	0.7900	0.8373 *	0.8893 *

* Indicates the best-fitting model.

**Table A5:** Coefficient of determination (R^2^) Hermia’s model. The wastewater concentration by UF process (Figure 13).

Feed	UF Time [min]	Complete Blocking		Standard Blocking		Intermediate Blocking		Cake Formation	
		UE10	UE50	UE10	UE50	UE10	UE50	UE10	UE50
blue + wax	0–160	0.7045	0.6240	0.7197	0.6516	0.7349	0.6822	0.7425 *	0.7646 *
	180–330	0.6900	0.6211	0.7276	0.6552	0.7643	0.6897	0.8306 *	0.7568 *
	350–480	0.6749	0.7756	0.6922	0.7935	0.7080	0.8088	0.7336 *	0.8308 *

* Indicates the best-fitting model.

**Table A6:** Coefficient of determination (R^2^) Hermia’s model. The UF process of real wastewater (Figure 14).

Feed	UF Time [min]	Complete Blocking		Standard Blocking		Intermediate Blocking		Cake Formation	
		UE10	UE50	UE10	UE50	UE10	UE50	UE10	UE50
real wastewaters	0–135	0.7932	0.7349	0.8310	0.7827	0.8655	0.8267	0.9220 *	0.8989 *

* Indicates the best-fitting model.
